# Determination of the Processes Driving the Acquisition of Immunity to Malaria Using a Mathematical Transmission Model

**DOI:** 10.1371/journal.pcbi.0030255

**Published:** 2007-12-28

**Authors:** João A. N Filipe, Eleanor M Riley, Christopher J Drakeley, Colin J Sutherland, Azra C Ghani

**Affiliations:** 1 Department of Epidemiology and Population Health, London School of Hygiene and Tropical Medicine, London, United Kingdom; 2 Department of Infectious and Tropical Diseases, London School of Hygiene and Tropical Medicine, London, United Kingdom; Utrecht University, The Netherlands

## Abstract

Acquisition of partially protective immunity is a dominant feature of the epidemiology of malaria among exposed individuals. The processes that determine the acquisition of immunity to clinical disease and to asymptomatic carriage of malaria parasites are poorly understood, in part because of a lack of validated immunological markers of protection. Using mathematical models, we seek to better understand the processes that determine observed epidemiological patterns. We have developed an age-structured mathematical model of malaria transmission in which acquired immunity can act in three ways (“immunity functions”): reducing the probability of clinical disease, speeding the clearance of parasites, and increasing tolerance to subpatent infections. Each immunity function was allowed to vary in efficacy depending on both age and malaria transmission intensity. The results were compared to age patterns of parasite prevalence and clinical disease in endemic settings in northeastern Tanzania and The Gambia. Two types of immune function were required to reproduce the epidemiological age-prevalence curves seen in the empirical data; a form of clinical immunity that reduces susceptibility to clinical disease and develops with age and exposure (with half-life of the order of five years or more) and a form of anti-parasite immunity which results in more rapid clearance of parasitaemia, is acquired later in life and is longer lasting (half-life of >20 y). The development of anti-parasite immunity better reproduced observed epidemiological patterns if it was dominated by age-dependent physiological processes rather than by the magnitude of exposure (provided some exposure occurs). Tolerance to subpatent infections was not required to explain the empirical data. The model comprising immunity to clinical disease which develops early in life and is exposure-dependent, and anti-parasite immunity which develops later in life and is not dependent on the magnitude of exposure, appears to best reproduce the pattern of parasite prevalence and clinical disease by age in different malaria transmission settings. Understanding the effector mechanisms underlying these two immune functions will assist in the design of transmission-reducing interventions against malaria.

## Author Summary

Whilst it is clear that natural immunity to malaria infection develops in those living in malaria-endemic regions of the world, the precise way in which it is acquired and the duration of immune memory are less-well-understood. We used a mathematical model that mimics malaria transmission between humans and mosquitoes in endemic settings to explore what epidemiological data, and in particular the prevalence of malaria in different aged individuals, can tell us about how immunity might develop. We explored three different parts of the transmission cycle at which immunity could act: 1) reducing the likelihood that an infected person develops symptomatic disease; 2) increasing the rate at which infection is cleared, and 3) increasing the duration of low-level (subpatent) infections that would continue to boost the immune system and hence protect against further disease. Our results show that the first two mechanisms together give rise to patterns of malaria by age group that are consistent with those observed in different malaria endemic settings in Africa. Our model also suggests that immunity to symptomatic disease lasts for at least five years, develops faster if there are higher levels of infection in the population, and increases with age. On the other hand, our model suggests that immunity that helps to clear infection lasts longer (20 years or more), develops later in life, and does not depend on the amount of transmission in the population.

## Introduction

Plasmodium falciparum malaria continues to be a major cause of human morbidity and mortality, especially in Africa, but varies greatly in endemicity across the continent and elsewhere [1]. The consequent variation in levels of acquired immunity and age-specific disease patterns complicates malaria epidemiology and means that control policies that are optimal for one setting are not easily translated to other settings. In highly endemic areas where clinical immunity develops rapidly [2]; there is concern that interventions which reduce transmission could also affect the development of immunity [3–6]. A delay in the acquisition of immunity beyond early life has the potential to change the spectrum of serious clinical symptoms [7,8] and the lifetime risk of disease [4].

While the processes that determine the acquisition of immunity to P. falciparum clearly impact on the epidemiology of the disease, they are complex and poorly understood due to the unclear relationship between immunological markers and functional immunity [9–11]. However, there is evidence to suggest that both clinical (anti-disease) immunity and anti-parasite immunity develop at different rates. For example, in people who emigrate from malaria endemic settings, clinical disease appears to emerge only in those who remain away for at least 3–5 y [7,12]. Furthermore, these emigrants also present clinically with lower parasite densities than those who travel from non-endemic areas, suggesting that an additional component of immunity that regulates parasite densities may be longer-lived. This hypothesis is also supported by analysis of age-stratified anti-malarial antibody seropositivity rates which gives estimates of half-lives that span decades [13]. There is also evidence to suggest that acquired immunity does not only depend on exposure but is also influenced independently by age. For example, there is evidence for an age-dependent exposure-independent maturation of the antibody response to malaria [14], and this may in part explain the observation that the proportion of severe malaria cases presenting with severe malarial anaemia is more closely associated with age than with transmission intensity [15,16]. Immune responses which affect subpatent parasitaemia may influence malaria transmission, but high rates of subpatent infection in high transmission areas suggest that acquired immune mechanisms capable of complete parasite clearance rarely develop in naturally exposed populations, so we allow for the possibility of subpatent tolerance.

Here we develop a mathematical model to better understand the impact of the development of immunity on observed epidemiological patterns, and also aspects of the immunology which might be inferred from the epidemiology such as time scales of acquisition and loss. Whilst a number of malaria transmission models have been developed in the past which incorporate immunity [17–25], each do so in different ways and hence make comparison between model structures difficult. In contrast, we systematically explore the impact of immune responses at different points of the host's natural history of infection which are then tested by comparing model output with epidemiological observations. Our results demonstrate that more than one type of age- and transmission intensity-specific response are necessary to predict malaria epidemiological patterns, in line with current immunological understanding [7,9,10,26].

## Results

### Immunity Functions Required To Reproduce Observed Age-Prevalence Patterns

We first developed an age-structured transmission model for malaria in which acquired immunity acts at three different stages of a host's history of infection: 1) susceptibility to symptomatic disease (severe and clinical cases) upon infection or re-infection, assuming susceptibility decreases with cumulative exposure to infectious bites (e.g., as a result of antibody-mediated strain-specific immunity); 2) natural recovery from asymptomatic to undetectable infection (i.e., effective clearance of parasites), which increases with cumulative exposure to infectious bites after a delay during childhood representing maturation of the immune system, 3) natural clearance of undetectable subpatent infection, assuming increased tolerance and slower clearance of such infection.

Each response, which we call an *immunity function*, is allowed to change with age and malaria transmission intensity (commonly expressed as the entomological inoculation (EIR)) and hence represents the acquisition and loss of immunity dependent upon exposure. The first two immunity functions incorporate a memory component (i.e., allow for gradual loss in the absence of reinfection) [27], whereas the final immunity function (associated with regulation of parasite density) is assumed independent of acquired immunity, as subpatent parasites (if any) are kept subpatent by an effective immune response.

**Figure 1:**
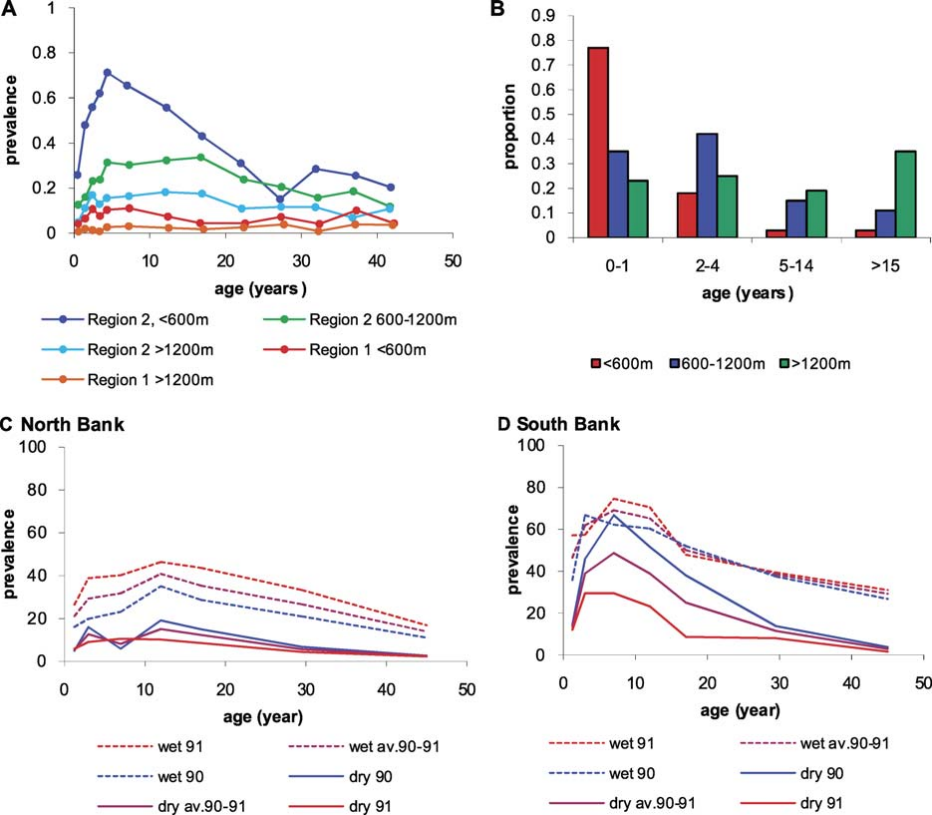
Observed Patterns of Parasitaemia and Clinical Episodes by Age in Areas and Seasons with Differing Transmission Intensity (A) Prevalence of parasitaemia by age, region, and altitude (<600 m, 600-1200 m, and >1200 m) from studies in Northern Tanzania. (B) Clinical episodes by age and altitude for region 2 (Usambara mountains) from severe malaria admissions to district, regional, and referral hospitals. (C,D) Prevalence of parasitaemia by age, year, and season (wet/dry) from North Bank (C) and South Bank (D) of River Gambia.

Figure 1A shows the patterns of parasitaemia and clinical disease by age observed in northern Tanzania. These data were collected from 24 villages at three different altitude levels (<600 m, 600-1200 m, and >1200 m) and in two different regions [28]. In one of the regions (region 2), estimates of malaria transmission intensity as measured by the EIR were also collected. These varied by altitude with the highest transmission intensity occurring at low altitude (56 infectious bites per person years (ibbpy), range 28–108 at <600 m, 3 ibppy, range 0.4–7.6 at 600-1200 m, and 0.12 ibppy, range 0.01–0.032 at >1200 m). Although these data were not available in the other region, the patterns of parasite prevalence by age and altitude are similar. Clinical data from severe malaria admissions to district, regional, and referral hospitals serving the Usambara mountain region (region 2) are shown in Figure 1B [15].

Figure 1C–1D shows the prevalence of parasiteamia by age in locations on the north bank and south bank of River Gambia, The Gambia [29]. Transmission in The Gambia is highly seasonal, and transmission intensity differs between the settings with higher intensity on the south bank. The estimates are presented separately for the dry and wet seasons, with higher prevalence observed during peak transmission in the wet season.

**Figure 2:**
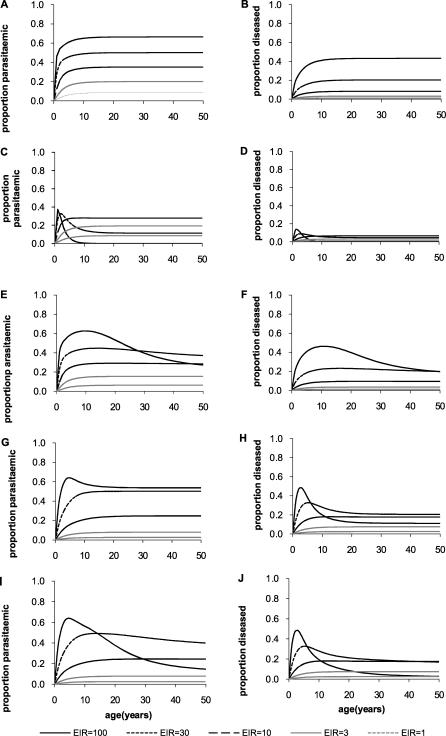
Predicted Relationship between Age and Parasitaemia or Clinical Disease for the Different Models of Immunity (A,B) No immunity; (C,D) immunity acting on clearance of subpatent parasites (immunity function 3); (E,F) immunity acting on clearance of detectable parasites (immunity function 2); (G,H) immunity acting on susceptibility to clinical disease (immunity function 1); (I,J) immunity acting on clearance of detectable parasites and susceptibility to clinical disease (immunity functions 1 and 2). Parameters are as shown in Table 1.

The corresponding patterns predicted by different versions of the model are shown in Figure 2. If the model does not incorporate immunity at any point, we observe a rise in the prevalence of parasitaemia or clinical disease which saturates at older ages (Figure 2A and 2B). This clearly does not match the decline in both parasitaemia and clinical disease at older ages observed in data (Figure 1). Allowing the model to incorporate immunity that results in increased persistence of subpatent infections (immunity function 3) gives rise to profiles that either peak too early in life and decay too rapidly at high EIRs or which saturate for low EIRs (Figure 2C and 2D). Allowing the model to incorporate immunity resulting in more rapid recovery from asymptomatic infections or symptomatic disease (immunity function 2) gives rise to patterns of parasitaemia that match those observed reasonably well. However, the patterns of symptomatic disease decay too slowly with age (Figure 2E–2F). Finally, allowing the model to incorporate immunity that reduces the proportion of infections that result in clinical disease (immunity function 1) results in patterns of clinical disease that closely match those observed in the data but fails to reproduce the decline in parasitaemia with age (Figure 2G–2H). Other discrepancies between the model predictions and observed patterns of parasitaemia and disease by EIR and inconsistencies in lifetime episodes were also observed for each immunity function (see Protocol S1).

**Table 1:**
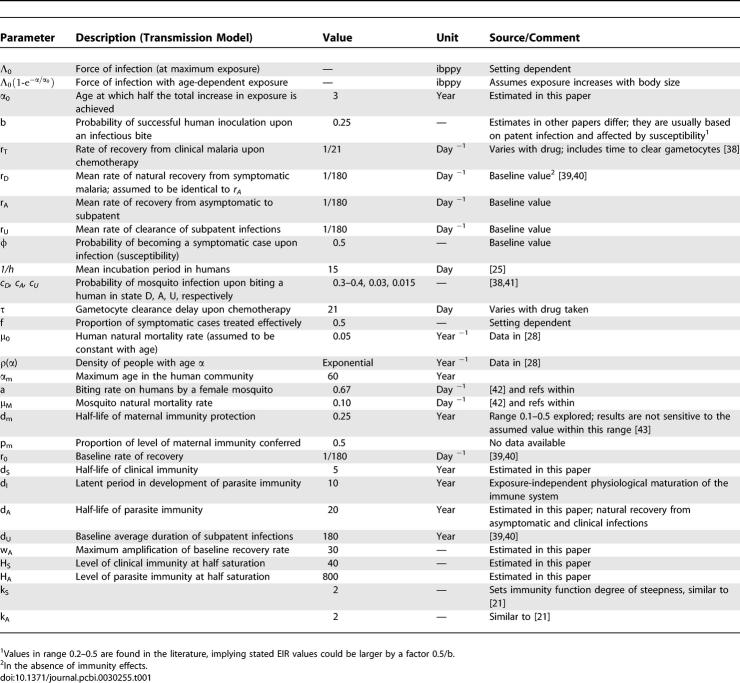
Summary of Model Parameters and Their Values

We next considered combining the different functions to identify which combination best reproduces the observed age-prevalence patterns in Figure 1. Combining immunity functions 1 and 2 (i.e., allowing a reduction in the proportion of infections that give rise to clinical disease and an increase in the rate of recovery from asymptomatic infection to subpatent infection) reproduces well the age-prevalence of parasitaemia and severe disease observed in the study data (Figure 2I and 2J). It also reproduces the observed decrease in clinical cases in older ages as the EIR is increased (see Protocol S1). Adding the third immunity function (increasing persistence of subpatent infection) results in patterns that more closely resemble those observed if this function alone drives immunity (Figure 2C and 2D) and therefore lessens the agreement between model predictions and observed data.

### Improved Model for the Impact of Immunity on Recovery from Asymptomatic Infection

**Figure 3:**
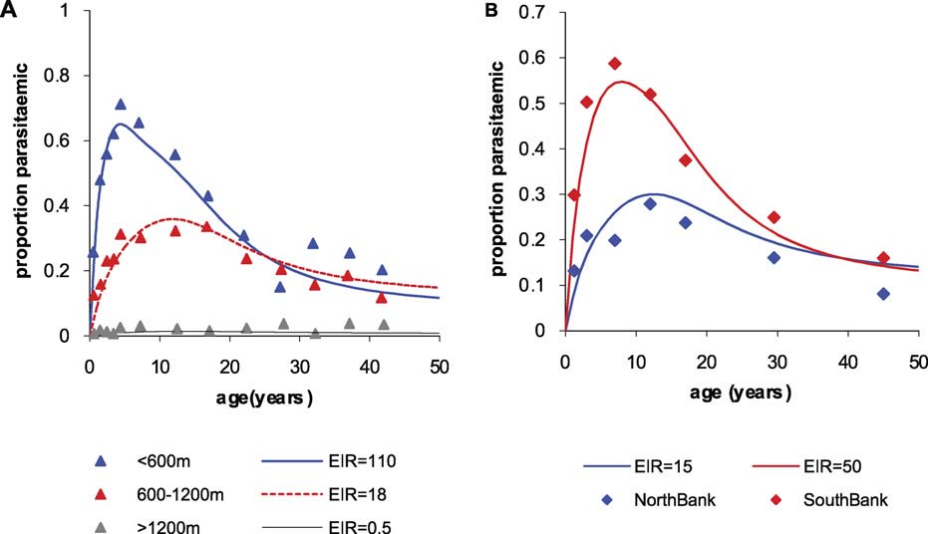
Predicted Relationship between Age and Parasitaemia at Different Levels of Transmission Intensity for the Model Incorporating Immunity Functions 1 and 2 and in Which Recovery from Infection Is Determined Solely by Age (A) Patterns predicted by the model compared to those observed in region 2 in Northern Tanzania by altitude. EIRs for the model are 110 for low altitude (measured EIR 28–108), 18 for medium altitude (measured EIR 0.4–7.6), and 0.5 for high altitude (measured EIR 0.01–0.32), percentage treated *f* = 50%. (B) Patterns predicted by the model compared to those observed on the north and south banks of the River Gambia. Model EIRs were 50 for the north bank and 15 for the south bank. Percentage treated *f* = 50%. All other parameters are as in Table 1. Our estimates of EIR are inversely proportional to the assumed value of parameter b; EIR estimates would be halved if we would assume *b* to be twice as large.

The age-prevalence patterns in Figure 2I and 2J resemble but do not exactly match those observed in data (Figure 1). There are many reasons for not expecting an exact match: estimates of EIR are imprecise, and quoted values are averages over surveys and locations within altitude ranges; there may be random variation and unaccounted factors, such as bias in data sampling among age groups; and parasite density and detection at a given age may differ among sites. However, we note that the model predicts age-parasitaemia curves which saturate with age for medium-to-low EIR, which is not observed in data. Adjusting parameters does not seem to alter this feature. However, if natural recovery from infection (e.g., from asymptomatic to subpatent) is solely determined by age (via physiological processes, provided there is exposure on which infection is conditional), we obtain patterns closer to those observed (Figure 3). This suggests that parasite immunity in non-naïve individuals may be controlled by physiological development rather than by the amount of natural exposure (provided there is exposure) [7–9,14,15,30].

### Patterns of Infectivity by Age

**Figure 4:**
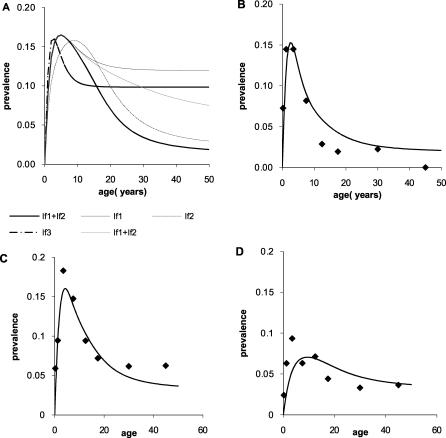
Observed and Predicted Patterns of Infectivity (Gametocytaemia) by Age in Tanzania and in The Gambia (A) Predicted infectivity by age from the model with different immunity functions. If1= immunity function 1 (susceptibility to clinical disease); If2 = immunity function 2 (clearance of detectable parasites); If3 = immunity function 3 (clearance of subpatent infection), If2* denotes EIR-independent version of If2. Parasitaemia is calculated in the model as symptomatic cases plus asymptomatic infections (D_H_+A_H_). All runs assume an annual EIR = 40 ibppy and that parameters are as before (Table 1), except c_D_ is adjusted (for If2 and If3) to make comparable the curves corresponding to different immunity function models. (B–D) Observed gametocytaemia by age from (B) the low altitude area of region 2 in Tanzania, (C) The Gambia south of the river bank, and (D) The Gambia north of the river bank. Parameters for the model are annual EIR = 110 (B), 50 (C), 15 (D), infectivity C_D_ = 0.3 as before (B,D), 0.4 (C), percentage treated *f* = 50%. All other parameters are as in Table 1.

An alternative way of testing the immunity functions (conditional on the remaining model structure and assumptions being valid) is to compare the predicted mean infectivity by age, which may be regarded as the probability of carrying gametocytes (although not all gametocyte carriers will be infectious), with the observed age-prevalence of gametocytes. The patterns predicted by our best model (incorporating immunity functions 1 and 2) closely match the patterns observed in northern Tanzania and The Gambia (Figure 4). Since the model parameters were fixed or fitted to asexual parasite data, these results are an independent test of the model's ability to reproduce observed epidemiological patterns.

### Duration of Clinical and Parasite Immunity

Our determined half-lives of clinical and parasite immunity were 5 y and 20 y, respectively. By varying these parameters, we explored whether patterns of age-prevalence can inform possible bounds for these parameters.

**Figure 5:**
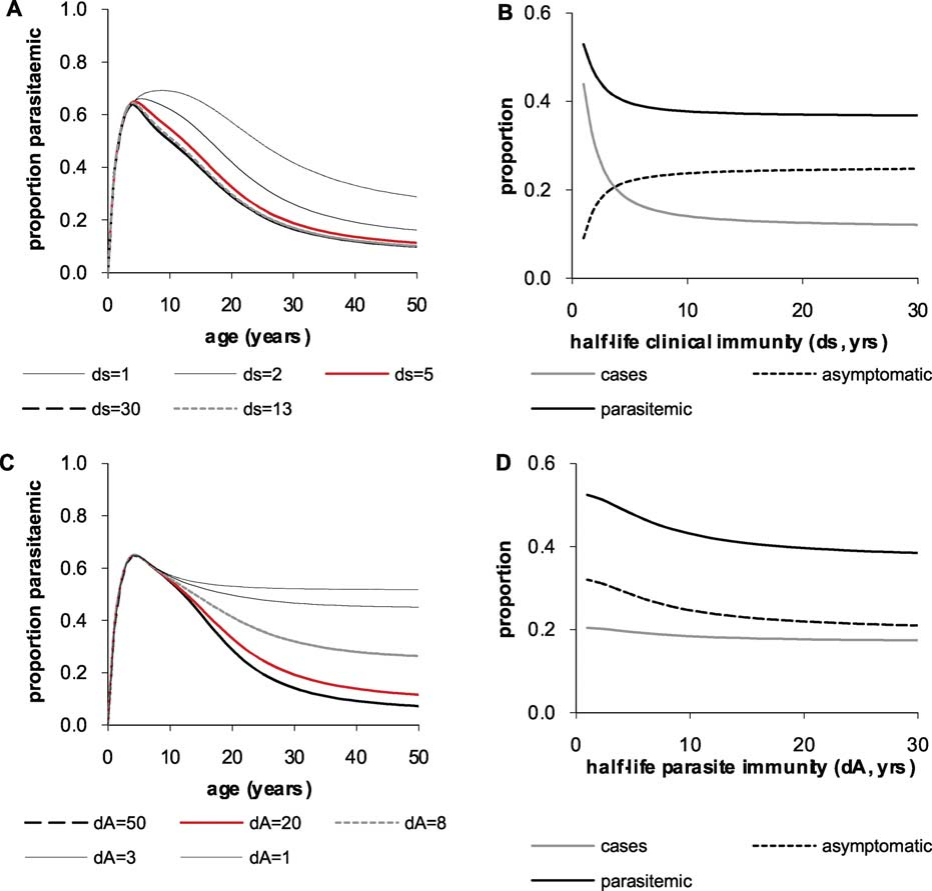
Sensitivity of the Relationship between Parasitaemia, Clinical Disease, and Age to Assumptions about the Duration of Acquired Immunity (A,B) Sensitivity to the duration of the immune response that reduces susceptibility to clinical disease where dS is the half-life; (A) shows the relationship between parasitaemia and age, and (B) shows the proportion of people predicted by the model to be symptomatic cases, have asymptomatic infections, and be parasitaemic (i.e., have patent infections) for different values of dS. Subpatent infections are not shown. For dS less than 5 y, the model predicts too high a proportion of all infections to be symptomatic cases rather than asymptomatic (B). (C,D) Sensitivity to the duration of the immune response that increases clearance of detectable parasites where dA is the half-life; (C) shows the relationship between parasitaemia and age, and (D) shows the proportion of people predicted by the model to be symptomatic cases, asymptomatic infections, and parasitaemic for different values of dA. For dA less than approximately 20 y, the model predicts that high levels of parasitaemia will persist into adulthood (C). Results are presented for an annual EIR of 110 ibppy. Similar patterns are obtained for lower EIR values.

Reducing the half-life for the duration of clinical immunity below 5 y results in a sharp increase in the proportion of all infections that are symptomatic cases and, in addition, results in less-pronounced age-prevalence peaks which begin to deviate from those observed in data. Increasing the duration of clinical immunity does not substantially change age-prevalence patterns but does have an impact on the proportion of infections that are symptomatic cases (Figure 5A and 5B).

Reducing the half-life for the duration of parasite immunity below 20 y similarly has an impact on the age-prevalence curves and at very low values (<10 y) gives rise to curves that saturate rather than decline at older ages. The proportion of infections that are asymptomatic and parasitaemic is also increased. However, increasing the duration of parasite immunity has little impact on either outcome (Figure 5C and 5D).

## Discussion

Our results demonstrate that, while distinct models can explain patterns of parasitaemia observed in individuals aged 0–5 y, in order to reproduce full age-prevalence patterns of parasitaemia and clinical disease observed in endemic malaria settings at least two distinct acquired immunity processes are required: 1) an early age (or early exposure) reduction in clinical susceptibility, and 2) a process of parasite immunity that increases the rate of natural recovery from infection and which develops substantially later in life (late childhood to adolescence). Adopting one of these processes in isolation does not reproduce observed patterns of age-prevalence of asexual parasitaemia, disease, and infectivity (gametocytaemia) across different endemicities (as measured by EIR). Moreover, while both clinical and parasite immunity were allowed to vary with age and EIR, the model in which natural recovery from infection (e.g., asymptomatic to subpatent) is determined solely by age better matches observed patterns than a model in which this is also determined by the intensity of exposure (EIR). This suggests parasite immunity in non-naïve individuals may be controlled by physiological processes rather than by amount of exposure (provided there is exposure). These findings agree with the current view that parasite immunity may require ageing to develop, but subsequently can persist without high antibody titres and therefore be maintained by occasional infrequent boosting [7–9]. Peaks in parasitaemia above 30 y of age present across endemic levels in eastern Tanzania might reflect malaria-HIV co-infection [31] and are not expected to be captured by the model.

Incorporating a prolonged duration of (subpatent) infections, i.e., continual reinfection that prolongs infection and boosts an immune response that allows parasitaemia to persist at subpatent levels, worsened the model predictions. However, we cannot exclude that an overall immune-modulated increase in duration of infections takes place, as suggested by recent hypotheses from within-host models [32] and in transmission models with fewer immunity components [17,18]. This is because the increase in duration of subpatent infection with increasing EIR could be weaker than considered by us. Furthermore, interpretation of this immunity function may depend on our model structure: we assume (via immunity functions 1 and 2) that a host returning to the noninfected state (S_H_) is likely to rapidly become asymptomatic with subpatent parasitaemia upon reexposure (i.e., is immune to symptomatic and to patent asymptomatic infection), tantamount to frequent subpatent infection but with recovery and reinfection modelled explicitly. Other models [17,18] assume persistent asymptomatic infection (though patency status may not be specified) which may be regarded as an implicit way of modelling this reinfection cycle.

Our model additionally allowed us to explore what age-prevalence patterns can tell us about the duration of clinical and parasite immunity. Our results suggest that clinical immunity has shorter memory (with a half-life of the order 5 y or more), while parasite immunity is effectively everlasting (with a half-life of 20 y or more after onset in adolescence). These durations are in line with evidence that migrating adults returning to endemic areas tend to become more sensitive to clinical attack but have lower parasite levels than children [8]; they are also in line with immunological studies in which one postulated mechanism of clinical immunity (antibodies to parasite phospholipids) has been shown to have a rather short half-life [33,34].

There are limitations in the epidemiological data that are available to inform model parameters. In particular, there are few and uncertain estimates of EIR by altitude range [28,35], as mentioned earlier. Furthermore, EIR estimates were not obtained from the same villages that were parasitologically surveyed, and the local history of interventions (which might affect the EIR) is not known. Therefore, discrepancies between observed and estimated EIR values are to be expected, especially in low-transmission areas where mosquito sampling is more difficult.

The model presented here clearly makes a number of simplifying assumptions. One of the main limitations is that the immunity functions, whilst generated based on current immunological understanding, could not be constrained by data. Further data on the way in which immunity develops and on the factors driving its development could help to refine these functions. The model also does not allow for partial immunity to reinfection, which would be relevant from the point of view of treating or vaccinating against pre-erythrocyte stages. While sterilising or partial pre-erythrocyte immunity are likely to be rare [7], it could be useful to extend the model to explore this possibility. Thirdly, we have not explicitly modelled the effects of parasite genetic diversity and have thus, strictly speaking, treated infections as monoclonal. However, the widely accepted hypothesis that immune development is regulated by antigenic variation and cumulative exposure to inoculations of differing parasite strains [20,22,26,32,36] is analogous to our definition of immunity levels in terms of cumulative exposure with finite memory. Our model is therefore consistent with theories in which immunity is strain-specific whilst integrating other aspects of acquired immunity development supported by cross-sectional data and current immunological understanding.

This age-structured malaria transmission model shares many features with existing models [17–25] but is novel in the way it combines epidemiological and immunological processes. Previous models have considered immune responses of types similar to those studied here (especially immunity that acts on the duration of asymptomatic and subpatent infections) [17,18,21,22,24], whilst others have represented acquired immunity through increased ability to reduce blood-stage parasite density [18,22,23,25].

Clearly, it is never possible to determine whether the structural assumptions behind any model represent the true processes generating the observed data, and it is likely that more complex model structures could also generate similar patterns. One alternative method that could be employed is to track parasite density rather than infection alone. Such an approach explicitly acknowledges variation in parasite load between individuals, and this variation may influence the development of immunity. However, such an approach also has its limitations. In particular, the distinction between disease and asymptomatic and subpatent infection requires definition of arbitrary parasite density thresholds for becoming diseased once infected and for detection by microscopy. Our assumption that susceptibility and recovery vary continuously via dependence on cumulative exposure is, however, analogous to the effect of immunity in bringing parasite density below such thresholds.

A second alternative method for incorporating immunity into mathematical models is to explicitly model strains and hence incorporate long-lasting strain-specific immunity. As noted above, our assumption that immunity develops with exposure and has finite memory essentially reproduces the patterns that would be obtained from such a model. The model does not imply that parasite density or strain-specific immunity are unimportant; as indeed there is strong evidence to support both playing a role in the development of immunity. Rather, our simpler model structure which implicitly incorporates these processes through immunity functions allows us to explore the timescales over which clinical and parasite immunity develop and are lost as well as the role of ageing and exposure on these functions.

Few previous models have been consistent in checking that they can reproduce the patterns of infection observed across a range of endemicities. By validating output against such patterns, we have sought to develop a model that is both informative about the impact of immunity on *falciparum* malaria epidemiology and also forms a solid basis with which to explore the impact of interventions. Having a robust framework which adequately captures the development of immunity with exposure and age is particularly important in exploring the impact of interventions such as insecticide treated nets (ITNs) and intermittent preventive therapy (IPT) in infants and children for which there is the potential to delay immunological development.

## Materials and Methods

### Mathematical transmission model.

**Figure 6:**
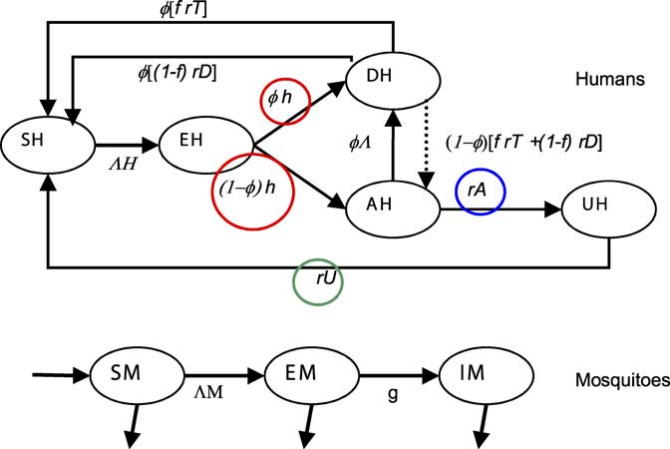
Schematic Illustration of the Full Transmission Model for Humans and Mosquitoes (without Explicit Ageing in Humans) States are shown in circles, and subscripts denote the population (H = humans, M = mosquitoes): susceptible S_H_/S_M,_ latent infection E_H_/E_M_, infected with symptomatic disease (severe and clinical cases) D_H_, asymptomatic patent infection A_H_, infected with undetectable (subpatent) parasite density U_H_, infectious mosquitoes I_M_. Λ_H_ /Λ_M_ is the force of infection on the human and mosquito populations, respectively, 1/h is the mean latent period in humans, 1/g the mean latent period in mosquitoes, φ is the proportion of human infections that develop disease, *f* the proportion of symptomatic cases that receive effective drug treatment, r_T_ the rate of recovery on treatment, r_D_ the rate of recovery without treatment, r_A_ the rate at which asymptomatic infections become subpatent, and r_U_ the rate at which subpatent infections are cleared. The coloured circles denote the stages at which acquired immunity can have an effect (modifying φ, r_A_, and r_U_). The parameters and their values are described in Table 1.

We model a human population with continuous age structure in which individuals of a given age can be in one of the following states: susceptible or not infected (S_H_), latent infection (E_H_), infected with symptomatic disease (including severe and clinical cases) (D_H_), asymptomatic with detectable parasites (A_H_), and asymptomatic infection with undetectable (subpatent) parasite density (U_H_). The main distinction between states D_H_ and A_H_ is that individuals in state A_H_ do not prompt treatment that leads to a change in infection state. The state U_H_ is included to account for the fact that measured parasitemia often decays with age, while highly sensitive parasite detection techniques suggest parasitemia continues increasing with age nearing 100% in highly endemic areas [37]. In tandem, we consider a mosquito population whose individuals can be susceptible (S_M_), exposed (latent) (E_M_), or infectious (I_M_). Figure 6 shows the transitions between states in each population (without displaying ageing). Susceptible humans move to latent infection at rate Λ, the force of infection on the human population. Individuals remain in this state for a mean duration 1/h (the mean latent period). A proportion φ develop disease whilst the remainder (1−φ) move to the asymptomatic infection category. A proportion *f* of symptomatic cases (D_H_) receive effective drug treatment and recover at rate r_T_, while the remaining cases recover naturally without treatment at rate r_D_. If clinical treatment or natural recovery is fully successful at removing parasites (with probability φ), the host returns to the susceptible state and otherwise moves to the asymptomatic state. Asymptomatic infections become subpatent at rate r_A_, and these subpatent infections are cleared at rate r_U_ with individuals returning to the susceptible state. Those in the asymptomatic state may additionally develop disease through superinfection at rate φΛ. Each human infection state, namely D_H_, A_H_, and U_H_, has a specific level of infectivity (transmission of mature gametocytes) to biting mosquitoes. The full equations for this model and further parameter definitions are given in Protocol S1.

Table 1 summarises variables, parameters, and the values used to generate the model outcomes presented in Results. Sensitivity analyses of model output to these parameters are presented in Protocol S1. In our analysis, we focus on results obtained once endemic levels are reached. Model outputs are generated by fixing the EIR or by fixing mosquito density (*m*) and calculating the EIR via the equations describing the mosquito section of the parasite's transmission cycle (see Protocol S1). We ignore any possible dependence of infectivity in the different infection states (D_H_,A_H_,U_H_) on age and EIR because this is currently less-well-understood [38]. For simplicity, we assume that the rate of natural recovery from clinical disease (r_D_) in the absence of treatment is identical to that from asymptomatic infection (r_A_), and that the rate of recovery of treated cases (r_T_) is determined by treatment only.

### Parameter estimation.

Unknown parameters (Table 1) were estimated by running the model over a wide range of plausible values and excluding values which lead to epidemiological patterns that clearly failed to visually match observed patterns. Our aim was to identify model structures and parameters values based on their ability to reproduce patterns and relationships. Given the many uncertainties in model structure, large number of parameters, and limited data available, it would have been very difficult to implement a more formal and rigorous statistical approach. Rather, we have focused on qualitative comparison and understanding. The sensitivity analyses to key parameters (in Protocol S1) give an idea of uncertainty and ranges of parameter values that might be expected on the basis of this model and datasets.

### Incorporation of acquired immunity.

To explore the impact that acquired immunity can have on patterns of age prevalence in endemic settings, we extend the basic transmission model above to incorporate immunity acting at three different stages of a host's history of infection. Mathematical details of the functions, described in brief below, are given in Protocol S1.

**Figure 7:**
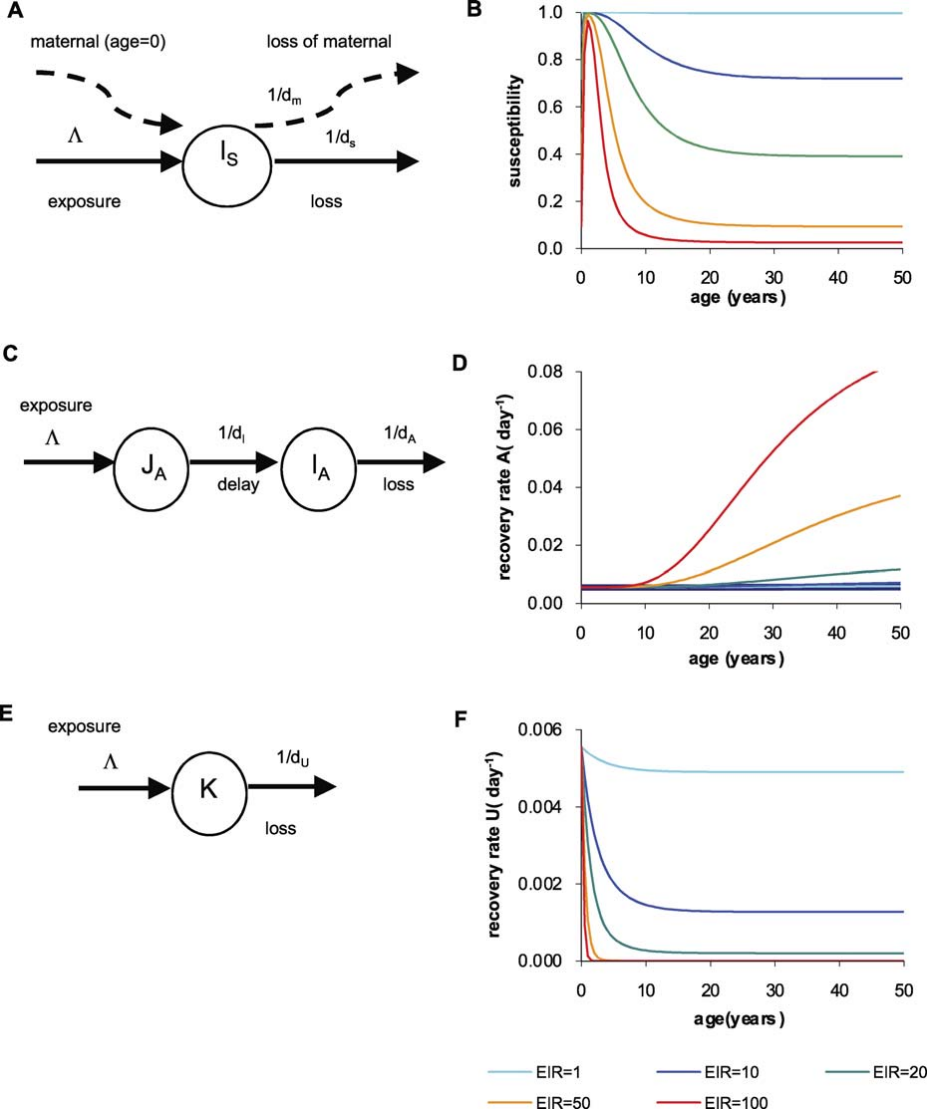
Immunity Functions That Act on: (A,B) the Susceptibility to Developing Clinical Disease; (C,D) the Clearance of Detectable Parasites, and (E,F) the Clearance of Subpatent Infection (A,C,E) Show schematically how each model assumes that immunity is developed (through exposure and/or age) and lost. (B,D,F) Show the resulting effect of these immunity levels on (B) susceptibility to clinical disease, (D) the rate of clearance of detectable parasites, and (F) the clearance of subpatent infection as people age and for five different transmission settings (identified by the EIR in ibppy). Further mathematical details are given in Protocol S1.

*1. Susceptibility to symptomatic disease, φ (immunity function 1).* We assume that individuals are born with maternally acquired immunity which is determined by the endemic level of disease and decays with a half-life d_M_. Following birth, clinical immunity accumulates due to exposure at a rate dependent on the force of infection in the population, Λ. This acquired immunity decays with a half-life d_S_. The schematic for this model is shown in Figure 7A. Susceptibility to symptomatic disease is then assumed to decrease in a nonlinear way as levels of clinical immunity increase. The overall dependence of susceptibility φ on age and EIR resulting from this model is shown in Figure 7B.

*2. Rate of natural recovery from asymptomatic to undetectable infection, r_A_ (immunity function 2).* The parasite immunity level associated with this response is similarly assumed to accumulate at a rate dependent on the force of infection in the population, Λ. The onset of parasite immunity is further assumed to have an age-related delay with mean d_l_, and any maternal immunity is lost during this period. Parasite immunity then decays with half-life d_A_. The schematic for this model is shown in Figure 7C. The recovery rate r_A_ is assumed to increase with levels of parasite immunity through a nonlinear function which saturates at higher levels of immunity. The overall dependence of recovery on age and EIR resulting from this model is shown in Figure 7D, where change with age follows from age-dependent exposure (see Protocol S1).

As an alternative, we also consider a model in which parasite immunity is determined only by age (given some exposure to infection) and not by EIR.

*3. Rate of natural clearance of undetectable infection, r_U_ (immunity function 3).* We assume that the duration of undetectable infection is boosted by continual reexposure and therefore not directly dependent on age. The onset of immunity is therefore dependent on the force of infection, Λ, and decays with half-life d_U_ as in previous models of superinfection [17,18]. A schematic for this is shown in Figure 7E and the resulting recovery rate as a function of the force of infection in Figure 7F.

## Supporting Information

Protocol S1 Mathematical Details and Sensitivity Analyses for Key Model Parameters

## Abbreviations

EIR: entomological inoculation rate
