# Supplementary material for: Determination of the Processes Driving the Acquisition of Immunity to Malaria Using a Mathematical Transmission Model
Source: PLoS Comput Biol. 2007 Dec 28;3(12):e255. doi: 10.1371/journal.pcbi.0030255 (PMC2230683; doi:10.1371/journal.pcbi.0030255)
Supplement: Protocol S1 [file pcbi.0030255.sd001.doc]

Determination of the processes driving the acquisition of immunity to malaria using a mathematical transmission model

J A N Filipe^1,*^, E M Riley^2^, C J Drakeley^2^, C J Sutherland^2^, A C Ghani^1^

^1^ Department of Epidemiology and Population Health and ^2^ Department of Infectious and Tropical Diseases, London School of Hygiene and Tropical Medicine, Keppel Street, London WC1E 7HT, United Kingdom

**Online Supplementary Material**

**Transmission Model**

We consider a human population with continuous age structure, in which individuals can be in one of the following states at age α and time *t* (with associated proportions in parenthesis): uninfected or susceptible (S_H_(α,t)), inoculated or exposed (E_H_(α,t)), infected with symptomatic disease (severe and clinicals cases) (D_H_(α,t)), with asymptomatic patent infection (A_H_(α,t)), and infected with undetectable (sub-patent) parasite density (U_H_(α,t)). A simultaneous mosquito population can be either susceptible (S_M_(t)), exposed (latent) (E_M_(t)) or infectious (I_M_(t)). The dynamics of infection transmission is given by the following set of partial differential equations:

$$\begin{matrix} \frac{\partial S_{H}}{\partial t}+\frac{\partial S_{H}}{\partial\alpha}=-\Lambda(\alpha) S_{H}+\varphi R_{D} D_{H}+r_{U}U_{H} \\ \frac{\partial E_{H}}{\partial t}+\frac{\partial E_{H}}{\partial\alpha}=\Lambda(\alpha) S_{H}-h E_{H} \\ \frac{\partial D_{H}}{\partial t}+\frac{\partial D_{H}}{\partial\alpha}=\varphi h E_{H}+\varphi\Lambda(\alpha) A_{H}-(R_{D} +\mu_{D})D_{H} \\ \frac{\partial A_{H}}{\partial t}+\frac{\partial A_{H}}{\partial\alpha}= (1-\varphi)h E_{H}+(1-\varphi) R_{D} D_{H}-\varphi\Lambda(\alpha) A_{H}-r_{A}A_{H} \\ \frac{\partial U_{H}}{\partial t}+\frac{\partial U_{H}}{\partial\alpha}=r_{A}A_{H}-r_{U}U_{H} \end{matrix}$$

where explicit age and time dependence is omitted. Here $\Lambda(\alpha)$(defined below) denotes the force of infection experienced by a person of age α, 1/*h* is the mean latent period, φ is the proportion that develop symptomatic disease, *f* is the proportion of symptomatic cases who receive effective drug treatment and recover at rate r_T_, and r_D_ is the natural recovery rate for those who do not receive treatment . r_A_ is the rate at which infections become subpatent and r_U_ is the rate at which subpatent infections are cleared. If clinical treatment or natural recovery is fully successful at removing parasites (with probability φ) the host returns to the susceptible state and otherwise moves to the asymptomatic state. Those in the asymptomatic state may additionally develop disease through superinfection at rate φΛ(α). $\text{R}_{D}=f r_{T} +(1-f) r_{D}$ incorporates the proportion treated (*f*) and µ_D_ is the rate of extra mortality due to severe disease. The density of people with given age α is defined by

$$\rho(\alpha)=\mu_{0}\frac{e^{-\mu_{0}\alpha}}{(1-e^{-\mu_{0}\alpha_{m}})}$$

which depends on human natural rate of mortality µ_0_, estimated in Figure A1, and on α_m_, the maximum age in the human population,. We assume the population is in demographic equilibrium (ρ(α) is time independent) and ignore extra mortality due to disease (µ_D_=0) because this model does not aim to study mortality and the proportion of fatal cases is comparatively small (although absolute numbers are very large [1])[[1]](#footnote-2). We also assume no differential natural mortality between states, so birth and natural mortality rates do not appear in the equations.

The human pre-patent infection state (E_H_) is short lived (~1-2 weeks) and may be ignored. A naïve host undergoes a disease cycle, S-E-D-S, before moving to the A state depending on how much the clinical susceptibility φ deviates from 1; this will involve a certain number of infections and occur at an average age that depends on EIR [2].

Vector dynamics are the same as in the Ross-Mac-Donald model [3] with force of infection Λ_M_ (see equation). The dynamics of the proportions S_M_, E_M_ and I_M_ of susceptible, exposed and infectious mosquitoes, respectively, are defined by the system of ordinary differential equations:

where μ_M_ is the mosquito mortality rate, τ_M_ is the parasite extrinsic latent period (assumed to be 10 days), and ψ=exp(-μ_M_τ_M_) is the probability that the mosquito survives the extrinsic latent period. We assume that the mosquito population is at equilibrium in relation to the human population dynamics:

$$I_{M}=\frac{\Lambda_{M}\Psi}{\Lambda_{M}+\mu_{M}}$$

where Λ_M_ is the force of infection experienced by mosquitoes:

$$\Lambda_{M}=a\int_{0}^{\alpha_{m}} \rho(\alpha)\left[c_{D}D(\alpha-\tau)+c_{A}A+c_{U}U\right]d\alpha$$

where *c_i_ (i=D,A,U)* is the human state-specific infectivity (probability of transmitting gametocytes to a biting mosquito), τ is the delay in clearance of gametocytes from peripheral blood circulation upon clearance of blood stages, and *ρ(α)* is the density of people by age as above.

The maximum force of infection acting on humans is then given by:

$$\Lambda_{H}=EIR b=(m a I_{M} )b$$

where *EIR* denotes the entomological inoculation rate, *b* is the probability of inoculation of a human upon a mosquito infectious bite, *m* is the density of mosquitoes per human, *a* the mosquito biting rate on humans and I_M_ the prevalence of infectious mosquitoes. The actual, age-dependent force of infection acting on humans is given by

$$\Lambda(\alpha)=\Lambda_{H}\text{(1-e}^{\text{-}\alpha/\alpha_{0}})$$

where α_0_ is the age of half increase in exposure. This form allows for a possible increase in exposure with age resulting, for example, from growing body surface. We found however that model output is not significantly sensitive to the range of values considered for α_0_ (a few months to a few years) and that the results are identical to those obtained assuming a constant force of infection equal to Λ_H_.

Tables S1 & S2 summarise variables, parameters and their values. We focus on temporal equilibrium dynamics in order to investigate the impact of hypothetical immunological responses on endemic epidemiological patterns (assuming no change in control interventions and thus constant EIR). Model outputs are generated by fixing the EIR or by fixing mosquito density (*m*) and calculating the EIR via the full parasite transmission cycle. In comparing the model with observations from seasonal settings we assume that equilibrium age-proportions are reached within the season and the EIR is an annual average. When EIR is fixed we do not need to make assumptions about infectivity *c_i_ (i=D,A,U)* unless we wish to predict entomological variables (*m* and *I_M_*).

**Table S1 –States and Variables in the Transmission M**odel.

| Variable | Description (transmission model) | comment |
| --- | --- | --- |
| α | human host age (units yr) |  |
| S(α,t) | proportion of people susceptible at age α & time t |  |
| E(α,t) | proportion of people inoculated with age α & time t |  |
| D(α,t) | proportion of people with severe disease at age α & time t |  |
| A(α,t) | proportion of people asymptomatic (but patent) at age α & time t |  |
| U(α,t) | proportion of people with sub-patent infection at age α & time t |  |
| I_M_ | proportion of mosquitoes that are infectious | See equation |
| Λ_M_ | average force of infection on mosquitoes | See equation |
| EIR | annual entomological inoculation rate (units ibppy[[2]](#footnote-3)) | variable if m is fixed;  typical range 0.5-100; See equation |
| *m* | density of female mosquitoes per human host | variable if EIR is fixed;  setting dependent; See equation |

**Table S2 – Parameters in the Transmission Model**

| parameter | Description (transmission model) | Value | Unit | Source/Comment |
| --- | --- | --- | --- | --- |
| *Λ_0_* | force of infection (at maximum exposure) | – | ibppy | setting dependent; see equation |
| $\Lambda_{H}\text{(1-e}^{\text{-}\alpha/\alpha_{0}})$ | force of infection with age-dependent exposure | – | ibppy | assumes exposure increases with body size |
| *α_0_* | age at which half the total increase in exposure is achieved | 3 | yr | Estimated in this paper |
| *b* | probability of successful human inoculation upon an infectious bite | 0.25 | – | Estimates in other papers differ; they are usually based on patent infection and affected by susceptibility[[3]](#footnote-4) |
| *r_T_* | rate of recovery from clinical malaria upon chemotherapy | 1/21 | day ^-1^ | varies with drug; includes time to clear gametocytes [4] |
| *r_D_* | mean rate of natural recovery from severe malaria; assumed to be identical to r_A_ | 1/180 | day ^-1^ | baseline value[[4]](#footnote-5) [5,6] |
| *r_A_* | mean rate of recovery from asymptomatic to sub-patent | 1/180 | day ^-1^ | baseline value; immunity function given by Equation |
| *r_U_* | mean rate of clearance of sub-patent infections | 1/180 | day ^-1^ | baseline value.; immunity function given by Equation |
| *φ* | probability of becoming a symptomatic case upon infection (susceptibility) | 0.5 | – | baseline value; immunity function given by Equation |
| *1/h* | mean incubation period in humans | 15 | day | [7] |
| *c_D_, c_A_, c_U_* | probability of mosquito infection upon biting a human in state D, A, U, respectively | 0.3-0.4, 0.03, 0.015 | – | [4,8] |
| *τ* | gametocyte clearance delay upon chemotherapy | 21 | day | varies with drug taken |
| *f* | proportion of symptomatic cases treated effectively | 0.5 | – | setting dependent |
| *µ_0_* | human natural mortality rate (assumed to be constant with age) | 0.05 | yr ^-1^ | data in [9] |
| *ρ(α)* | density of people with age α | exponential | yr ^-1^ | Equation and data [9] |
| *α_m_* | maximum age in the human community | 60 | yr |  |
| *a* | biting rate on humans by a female mosquito | 0.67 | day ^-1^ | [10] and refs within |
| *µ_M_* | mosquito natural mortality rate | 0.10 | day ^-1^ | [10] and refs within |

**Immunity Functions**

*1. Susceptibility*

The dynamics of the clinical immunity level, which controls the susceptibility response, has a value at birth conferred vertically with half-life *d_m_*, and accumulates due to exposure with incidence equal to the force of infection and half-life *d_s_*. These dynamics are described by the partial differential equation:

$$\frac{\partial I_{s}}{\partial t}+\frac{\partial I_{s}}{\partial\alpha}=\Lambda(\alpha) -(1/d_{s}) I_{s}+(1/d_{s}-1/d_{m}) I_{s}(t,0) e^{-\alpha/d_{m}}$$

where I_s_(t,0) is the innate level of immunity at birth which is a proportion *p_m_* of the maternal immunity level assumed to be the maximum immunity level attained with age (this saturates at 15-20 yrs and is ~Λ(α_max_) d_s_). For time-independent EIR (in absence of changes due to interventions, seasonal or climatic variation) this can be solved to obtain:

$$I_{s}(\alpha)=\Lambda(\alpha_{\max}\text{)d}_{\text{s}}\text{(1+}\frac{\alpha_{0}\text{e}^{\text{-}\alpha\text{/}\alpha_{0}}-\text{t}_{\text{s}}\text{e}^{\text{-}\alpha\text{/d}_{\text{s}}}}{\text{d}_{\text{s}}-\alpha_{0}}\text{)+}I_{s}(0) e^{-\alpha/d_{m}}$$

The susceptibility response is assumed to be a nonlinear decreasing function of the immunity level I_s_(t,α):

The dependence of φ on age and EIR is shown in Figure 2, and on immunity level is shown in Figure S1. The parameters are described in Table S3.

*2. Recovery from asymptomatic infection*.

The parasite immunity level, which is associated with this response, accumulates due to exposure with incidence equal to the force of infection, has an average delay *d_l_* related to age development, and half-life *d_s_*; vertically conferred immunity is assumed to be lost during the delay; these dynamics are described by

with J_A_ the latent immunity level and no surviving innate immunity (i.e. J_A_ and I_A_=0 at birth). For time-independent EIR this system of equations can be solved explicitly. The recovery rate response is assumed to be a saturating increasing function of the immunity level I_A_(t,α):

$$r_{A}(I_{A})=r_{0}\left[1+(w_{A}-1)\frac{(I_{A}/H_{A})^{k_{A}}}{1+(I_{A}/H_{A})^{k_{A}}}\right]$$

Parameters are described in Table S3. The dependence of *r_A_* on age and EIR is shown in Figure 2, and on immunity level is shown in Figure S1.

An alternative model for this response representing changes with age only (given there is exposure) is to replace the EIR-dependent magnitude of the incidence, Λ_H_ (see equations and ), with a fixed value (e.g. maximum in the range). This is identified in the main text as the optimal model.

Functional forms and resemble those in [11], but in there no distinction is made between infection and disease and the dynamics of the immunity levels are not made explicit; there is also some relation to assumptions in [7], but those are applied to log parasite density.

*3. Clearance of sub-patent infections.*

We assume the duration of this immune-protected state in boosted by continued re-exposure and not directly dependent on age (as a host reaching this state will have gone through a long period of exposure). We use the well-know ‘superinfection’ recovery rate form [12,13]

$$r_{U}(\Lambda(\alpha))=\frac{\Lambda(\alpha)}{e^{\Lambda(\alpha)d_{U}}-1}$$

Figure S1: Immune responses as functions of the associated immunity level. For immunity functions 1 and 2 the relative immunity level is in units of H_S_ and H_A_ respectively.

**Table S3 – Parameters for the** immunity functions.

| parameter | description (immunity functions) | value | unit | source/comment |
| --- | --- | --- | --- | --- |
| *d_m_* | half-life of maternal immunity protection | 0.25 | yr | Range 0.1-0.5 was explored; results are not sensitive to the assumed value within this range [14] |
| *p_m_* | proportion of level of maternal immunity conferred | 0.5 | – | No data available |
| *r_0_* | baseline rate of recovery | 1/180 | day^-1^ | [5,6] |
| *d_S_* | half-life of clinical immunity | 5 | yr | Estimated in this paper |
| *d_l_* | latent period in development of parasite immunity | 10 | yr | Estimated in this paper; exposure-independent physiological maturation of the immune system |
| *d_A_* | half-life of parasite immunity | 20 | yr | Estimated in this paper; natural recovery from asymptomatic & clinical infections |
| *d_U_* | baseline average duration of sub-patent infections | 180 | yr | [5,6] |
| *w_A_* | maximum amplification of baseline recovery rate | 30 | – | Estimated in this paper |
| *H_S_* | level of clinical immunity at half saturation | 40 | – | Estimated in this paper |
| *H_A_* | level of parasite immunity at half saturation | 800 | – | Estimated in this paper |
| *k_S_* |  | 2 | – | Sets immunity function degree of steepness, similar to [11] |
| *k_A_* |  | 2 | – | similar to [11] |

**Table S4 – Main types and determining factors of the immune responses (chosen model).**

| Determining factor \ immunity function | Susceptibility (If1) | Parasite clearance in asymptomatic (If2) | Parasite clearance in non-patent (If3) |
| --- | --- | --- | --- |
| Exposure | YES | NO | YES |
| Age or time | YES | YES | NO |
| Memory (limited duration) | YES | YES | N/A |

**Parasitological and Epidemiological Data**

*Tanzanian Parasitological Data*

Individual measurements of asexual parasitemia and gametocyteamia were available from 24 villages in 2 regions and 6 altitude transects (<600m, 600-1200m, >1200m) in NE Tanzania [9] pooled from 2 surveys in the dry and wet seasons . The age distribution of the population is shown in Figure S2. The natural mortality rate μ_0_ is estimated from these data.

Figure S2: Age-distribution of the population (all villages) in the NE Tanzanian study [9]. The line represents a least-square fit assuming constant rate (type 2) mortality, giving μ_0_ ~0.054 1/y. Similar estimates are obtained if the analysis is restricted within a region and altitude range.

*Gambian Parasitological Data*

Asexual parasite prevalence and gametocyte prevalence (an index of infectiousness) by age was obtained at two locations south and north of the River Gambia. Data were obtained in 2 seasons (wet and dry) and from two survey years 1990 and 1991 (see main text). Further details are given in [15].

*Tanzanian Clinical Data*

Data on severe malaria admissions from district, regional and referral hospitals serving hospitals from the Usambara mountains in NE Tanzania is given in reference [16]. The original data are presented as the proportion, P_i_, of severe malaria cases by age group among all severe malaria admissions in each altitude range. To convert overall proportions into patterns of age-prevalence, p_i_, we use the relationship for each age group,

$$P_{i}=\frac{\rho_{i} p_{i}}{\sum_{j} \rho_{j} p_{j}}=\frac{\rho_{i} p_{i}}{P_{D}}$$

where

is the proportion of the population in age group i and P_D_ is the overall prevalence of cases in the specific altitude range, giving

$$p_{i}=\frac{P_{D} }{\rho_{i}}P_{i}$$

The local prevalence of severe cases, *P_D_*, is unknown as it depends on local demography, access to treatment etc, but the pattern of age-disease can still be determined up to this altitude-specific magnitude factor. We chose to divide case prevalence in each age group by case prevalence in the first age group (age 0-1 years), giving the patterns in Figure 3b.

**Immune functions and predicted average proportions and lifetime episodes**

*Figure S3* shows the population average proportions and lifetime episodes respectively predicted by models with different models for immune function. As for the patterns of age-prevalence shown in the main text, models with no immune response fail to reproduce expected patterns. Discrepant patterns noted below are also observed for immunity functions 2 and 3.

*Model with variable recovery of undetectable infections (Immunity function 3):* The average proportion of asymptomatic infection decays with EIR (whereas we expect an increase) and the proportion undetectable increases too much with EIR (Figure S3 c-d). The intensity of these effects is reduced if we assume a shorter duration of the U state. This type of immune response has been used in several malaria transmission models, e.g. [12,13].

*Model with variable recovery of asymptomatic infections (Immunity function 2):* The average proportion that are asymptomatic decays with EIR (whereas we expect an increase) and the proportion diseased increases with EIR (Figure S3c) while we expect the opposite. A similar erroneous pattern is observed for lifetime disease episodes (Figure S3d).

*Model with variable susceptibility (Immunity function 1):* Here the average proportions in all classes and the number of lifetime episodes of disease and asymptomatic infection seem plausible (Figure S3e-f).

*Model with combined immune responses:* Combining different immunity functions, as for age-prevalence patterns, improves the fit. The model combining variable susceptibility and asymptomatic recovery (immunity functions 1 and 2) predicts a continued decrease in cases at high EIR as would be expected (Figure S3i-j). The clinical data, although approximate, is roughly consistent with the model. The average proportions and lifetime episodes of symptomatic disease and asymptomatic infection are as for the model with variable susceptibility alone but the proportion undetected increases with EIR, which seems more plausible (Figure S3g-h).Therefore it appears necessary to include these two immunity-modulated responses in order to capture both patterns of parasitemia and disease.

Figure S3: Population average proportions and lifelong episodes predicted by models assuming different immune responses. Immunity function (If) 3 (a-b), If2 (c-d), If 1 (e-f), If 1 & 2 (g-h), If 1 & 2 +alternative model for r_A_ (i-j).

**Sensitivity analyses**

In this section we present brief results from sensitivity analyses on key parameters. We distinguish between two model versions: the first with fixed EIR (presented in the main text) and the second with fixed vector density (*m*) in which human variables depend on the full cycle of parasite transmission. The distinction is important if *m* is a determining factor (and EIR is a dependent variable) or human infectivity parameters change, but not when other parameters vary. Fixing *m* is necessary when there are interventions which can vary EIR but not mosquito density.

*Proportion of cases treated, f.*

This parameter depends on local health policy and access to primary health care facilities [17]. With no specific knowledge we have so far assumed *f*=50%, but now vary *f* with other parameters unchanged.

For a fixed annual EIR=40 the proportion of symptomatic cases decreases and the proportion of asymptomatic and undetected infections increase and saturate as treatment is varied from none (*f*=0) to all cases (*f*=1) as expected (Figure S4a-b). As the proportion of cases treated is increased, mosquito density *m* increases from 2 to 9 and the proportion of mosquitoes that are infectious consequently drops from 8% to 2% (not shown) in line with typical field values.

If instead the mosquito density is fixed at *m*=10 (equivalent to an EIR of approximately 40 when *f*=0.5) the average proportion in each infection state decreases as the proportion of cases treated increases as expected. Lifetime episodes of disease double while lifetime asymptomatic infections and EIR drop sharply as *f* is increased (Figure S4c-d). For *m*=5 (equivalent to an EIR of approximately 15) there is elimination if more than 70% of cases are treated (Figure S4e-f).

Figure S4: Effect of proportion of symptomatic cases treated on population proportions in each infected state and lifetime episodes (f=0.5 was assumed in previous results). (a-b): fixed EIR=40, but results do not vary much with EIR. (c-d): fixed m=10. (e-f): fixed m=5.

*Mosquito density, m.*

As mosquito density is increased (keeping *f*=0.5), the average proportion diseased decreases, the proportion undetected increases and the proportions parasitemic and asymptomatic saturate as would be expected. Lifetime episodes of disease drop by over 50%, lifetime asymptomatic infections saturate and EIR increases linearly with increasing *m* (Figure S5a-b).

*Infectivity of asymptomatic infections, c_A_.*

Altering the values of infectivity of asymptomatic and undetected infections (keeping the first twice as large as the second) has little effect on the proportions in each infection state because of immunity trade-off effects. Nevertheless, lifetime disease episodes increase and EIR decreases significantly with increasing c_A_ (Figure S5i-j). These results are shown for *m*=5. For *m*=2.5 proportions of infections and lifetime episodes are similar but generally lower (not shown).

*Infectious period of treated infections.*

Varying the infectious period (i.e. gametocytemia) following successful treatment between 2-4 weeks (3 weeks was the value used) produces roughly similar results. However, a period below 2 weeks would yield substantially lower predictions of infection and disease and even optimistic elimination (if it is assumed below 10 days) (Figure S5e-f). These results are for *m*=5 which correspond to EIR~20 when a 3 week infectious period is assumed.

*Duration of untreated infections*.

These results are analogous to the previous. A maximum natural infectious period (in absence of a reduction due to immunity) of 2-6 months (6 months was the value used) would produce somewhat similar results. However, a period below 1-2 months would yield substantially lower predictions of infection and disease and even optimistic elimination (below 3-4 weeks) (Figure S5g-h). These results are for *m*=5 which corresponds to EIR~20 for a 3-6 month duration for untreated infections.

Figure S5: Sensitivity analysis. Change in proportions in each infected state and lifetime episodes as parameter depart from values assumed in Tables 1-3 (and EIR is no longer fixed): (a-b) mosquito density m; (c-d) proportion of infectivity of asymptomatic relative to diseased (with undetected infections half as infectious); (e-f) infectious period of treated cases; (g-h) duration of untreated infections in absence of immunity. Parameters: m=10 is fixed corresponding to EIR~40 ibppy; 10% infectivity of asymptomatic relative to cases; 3 week infectious period upon treatment; 3-6 months infectious period upon natural naïve recovery.

**Memory of acquired immunity.**

*Clinical immunity (susceptibility response).*

The value set for the half-life of clinical immunity (5 yrs) is a minimum below which prevalence-age patterns are not well reproduced and overall proportions of parasitemia and disease are significantly changed. Above the set value, age patterns are not much affected but overall proportions are (Figure S6). This suggests that, all else being equal, average duration of clinical immunity could be slightly less or slightly more but is unlikely to be significantly different from 5 yrs.

Figure S6: Memory of clinical immunity, sensitivity analysis. Change in age-parasitaemia and proportion in each infected state as duration of clinical immunity half-life (t_c_) increases. Annual EIR=110 ibppy (a, c), 18 (b, d).

*Parasite immunity (clearance response).*

The value set for the half-life of parasite immunity (20 yrs) is a minimum below which prevalence-age patterns are not reproduced and overall proportions of parasitemia and disease are significantly changed. Above the set value, age patterns and overall proportions are not changed (). This suggests that, all else being equal, duration of parasite immunity is unlikely to be less but could be larger than 20 yrs.

Figure S7: Memory of parasite immunity, sensitivity analysis. Change in age-parasitaemia and proportion in each infected state as duration of parasite immunity half- life (t_b_) increases. Annual EIR=110 (a, c), 18 (b, d).

References

1. Greenwood BM, Bojang K, Whitty CJM, Targett GAT (2005) Malaria. Lancet 365: 1487-1498.

2. Baird JK (1995) Host Age as a Determinant of Naturally Acquired-Immunity to Plasmodium-Falciparum. Parasitology Today 11: 105-111.

3. Koella JC (1991) On the Use of Mathematical-Models of Malaria Transmission. Acta Tropica 49: 1-25.

4. Drakeley C, Sutherland C, Bouserna JT, Sauerwein RW, Targett GAT (2006) The epidemiology of Plasmodium falciparum gametocytes: weapons of mass dispersion. Trends in Parasitology 22: 424-430.

5. Miller MJ (1958) Observations on the natural history of malaria in the semi-resistant West African. Trans R Soc Trop Med Hyg 52: 152-168.

6. Jeffery GM, Eyles DE (1955) Infectivity to mosquitoes of Plasmodium falciparum. Am J Trop Med Hyg.

7. Maire N, Smith T, Ross A, Owusu-Agyei S, Dietz K, et al. (2006) A model for natural immunity to asexual blood stages of Plasmodium falciparum malaria in endemic areas. American Journal of Tropical Medicine and Hygiene 75: 19-31.

8. Alves FP, Gil LH, Marrelli MT, Ribolla PE, Camargo EP, et al. (2005) Asymptomatic carriers of Plasmodium spp. as infection source for malaria vector mosquitoes in the Brazilian Amazon. J Med Entomol 42: 777-779.

9. Drakeley CJ, Carneiro I, Reyburn H, Malima R, Lusingu JPA, et al. (2005) Altitude-dependent and -independent variations in Plasmodium falciparum prevalence in northeastern Tanzania. Journal of Infectious Diseases 191: 1589-1598.

10. Gu WD, Mbogo CM, Githure JI, Regens JL, Killeen GF, et al. (2003) Low recovery rates stabilize malaria endemicity in areas of low transmission in coastal Kenya. Acta Tropica 86: 71-81.

11. Gu WD, Killeen GF, Mbogo CM, Regens JL, Githure JI, et al. (2003) An individual-based model of Plasmodium falciparum malaria transmission on the coast of Kenya. Transactions of the Royal Society of Tropical Medicine and Hygiene 97: 43-50.

12. Dietz K, Molineau.L, Thomas A (1974) Malaria Model Tested in African Savannah. Bulletin of the World Health Organization 50: 347-357.

13. Aron JL (1988) Mathematical-Modeling of Immunity to Malaria. Mathematical Biosciences 90: 385-396.

14. Riley EM, Wagner GE, Akanmori BD, Koram KA (2001) Do maternally acquired antibodies protect infants from malaria infection? Parasite Immunology 23: 51-59.

15. Drakeley CJ, Akim NIJ, Sauerwein RW, Greenwood BM, Targett GAT (2000) Estimates of the infectious reservoir of Plasmodium falciparum malaria in The Gambia and in Tanzania. Transactions of the Royal Society of Tropical Medicine and Hygiene 94: 472-476.

16. Reyburn H, Mbatia R, Drakeley C, Bruce J, Carneiro I, et al. (2005) Association of transmission intensity and age with clinical manifestations and case fatality of severe Plasmodium falciparum malaria. Jama-Journal of the American Medical Association 293: 1461-1470.

17. Barnes KI, Durrheim DN, Little F, Jackson A, Mehta U, et al. (2005) Effect of artemether-lumefantrine policy and improved vector control on malaria burden in KwaZulu-Natal, South Africa. Plos Medicine 2: 1123-1134.

1. Demographic equilibrium in the presence of extra mortality would require extra susceptible newborns. [↑](#footnote-ref-2)
2. Infectious bites per person per year (ibppy) [↑](#footnote-ref-3)
3. Values in range 0.2-0.5 are found in the literature implying stated EIR values could be larger by a factor 0.5/b. [↑](#footnote-ref-4)
4. In the absence of immunity effects. [↑](#footnote-ref-5)
